# Fruit Smoothies Enriched in a Honeysuckle Berry Extract—An Innovative Product with Health-Promoting Properties

**DOI:** 10.3390/foods12193667

**Published:** 2023-10-05

**Authors:** Marta Waszkiewicz, Anna Sokół-Łętowska, Aleksandra Pałczyńska, Alicja Z. Kucharska

**Affiliations:** Department of Fruit, Vegetable and Plant Nutraceutical Technology, Wrocław University of Environmental and Life Sciences, Chełmońskiego 37, 51-630 Wrocław, Poland

**Keywords:** food bioactives, polyphenols, iridoids, functional food, biological activity, antioxidant activity

## Abstract

Smoothies are claimed to be an effective way of promoting fruit and vegetable consumption. They are a rich source of bioactive compounds and provide numerous health benefits. Strawberries and apples are among the most popular smoothie ingredients. Additionally, chokeberry presents antibacterial, antiviral and anti-inflammatory properties. Another interesting fruit with a wide range of health benefits is the honeysuckle berry. In this study, a dry extract from the mentioned fruit was combined to produce a smoothie enriched in bioactive compounds of unique health-promoting properties. The smoothies were rich in anthocyanins, flavonols, phenolic acids, flavan-3-ols and iridoids. Smoothies with higher concentrations of a polyphenol-iridoid honeysuckle berry extract (0.50%) were the products of a greater content of bioactive compounds and higher antioxidant activity compared to those with no extract or a lower amount (0.25%). However, the sensory evaluation showed that, according to customers, the least attractive smoothies are those with the greatest amounts of the honeysuckle berry extract. Therefore, the correct balance between taste and bioactivity should be sought in order to obtain an innovative product showing characteristics of functional food.

## 1. Introduction

Functional food is attracting more and more attention mainly due to increased consumer awareness in terms of food quality and widely promoted healthy lifestyle as well as attracting attention to little-known plants of exceptional health properties [1]. Functional food has a beneficial effect on one or more functions in the body, such as nutritional effects, improving the state of health or reducing the risk of disease [2]. The available data indicate that consumption of products rich in bioactive compounds is connected with a decreasing risk of chronic diseases, such as obesity, tumours, and metabolic syndrome [3]. Smoothies are considered an excellent and convenient way to promote consumption of vegetables and fruit [4]. Fruit-based smoothies constitute the largest part of the smoothies industry and are also the fastest-growing branch [5]. It has been proved that smoothies contain more dietary fibre, vitamin C and other antioxidant compounds than fruit juice [6,7]. Moreover, they are rich in polyphenols able to scavenge reactive oxygen species and showing high health potential [8]. Numerous commercial smoothies are based on bananas, apples, and also with the addition of red fruit like strawberries, blackberries, raspberries [9]. As far as bioactivity is concerned, chokeberries, bilberries, cranberries and blackcurrants contain the most bioactive compounds [1]. Meanwhile, the honeysuckle berry is another fruit known to exhibit high health-promoting properties but is less known and thus undervalued. In this study, strawberries, chokeberries, apples and honeysuckle berries have been chosen for smoothie production.

Strawberry is a popular fruit commonly consumed around the world due to its attractive flavour and aroma. Moreover, it is a rich source of antioxidants [10]. Total phenolic compound content consists of 41% anthocyanins, 28% flavan-3-ols, 14% ellagitannins, 13% cinnamic acid conjugates, 3% flavonols, and 1% ellagic acid conjugates. The most important compound present in strawberries in terms of health benefits is ellagic acid, reaching levels in the range of 4.056.4 mg/100 g [11,12]. Strawberries also contain a wide range of flavonols, mainly quercetin-3-glucoronide. Moreover, the fruit is a source of B vitamins (B1, B2, B3, B6) as well as vitamins A, E [11,12,13,14]. The content of vitamin C in this fruit exceeds that in citrus fruit and is found in the range of 40–100 mg/100 g. Strawberries are also rich in folic acid and minerals such as phosphorus, calcium, and magnesium.

Chokeberries are a rich source of vitamins B_2_, B_6_, E, P, and PP [15]. Their health-promoting properties are mainly associated with their high polyphenol content [16]. Polyphenols are responsible for most of the health benefits of the fruit [17]. Anthocyanins decrease the side effects of antitumor treatment [18,19] and show a positive influence on the cardiovascular system; they also have antibacterial, antiviral and anti-inflammatory properties [20]. Research has proved that the antiviral properties of chokeberry are mainly associated with the presence of isoquercetin, ferulic acid and kaempferol.

An apple is characterized by a high content of flavonoids, including quercetin, catechin and chlorogenic acid [21]. The popular saying “an apple a day keeps the doctor away” has a scientific basis, as it has been proved that consumption of apples reduces the risk of cancer, asthma, diabetes and cardiovascular disease. The antioxidant activity of 100 g of apples is equal to that of 1500 mg of vitamin C, though such a portion contains only 5.7 mg of the vitamin [22].

A honeysuckle berry fruit is used after heavy metal or drug poisoning due to its detoxification properties [23]. Additionally, honeysuckle berries reduce the adhesion of bacteria to the urinary tract, thus counteracting the infections [24]. Anthocyanins found in a honeysuckle berry show an ability to regulate tissue microcirculation, which is an important factor in treating eye disorders. It has been reported that consumption of a honeysuckle berry extract reduces the level of proinflammatory factors such as nitric oxide, tumor necrosis factor-α, interleukin-1β, prostaglandin E2 and cyclooxygenase-2 [25,26]. A honeysuckle berry extract enhances the activity of antioxidant enzymes such as superoxide dismutase and reduces products of lipid peroxidation. The extract is also able to inhibit transcription factors involved in inflammation that possibly lead to carcinogenesis (nuclear factor-κB cytokines, lipoxygenases, nitric oxide synthase) [27]. Apart from polyphenol compounds, iridoids, which are rarely present in fruit, have been found in honeysuckle berries [28]. Iridoids demonstrate properties of endogenous neurotrophic factors, showing a beneficial potential in treating neurodegenerative diseases [29]. They can generally be used for intracerebral targeting, as they can pass the blood–brain barrier [30]. Moreover, iridoids show hepatoprotective and antitumor effects, as well as hypolipidemic and hypoglycaemic activities. A honeysuckle berry (*Lonicera caerulea* L.) has already been considered by Japanese Ainu aborigines as an “elixir of life”, and its healing properties have been applied in traditional folk medicine mainly to treat hypertension, anaemia, glaucoma, and osteoporosis [31,32,33]. A recent study revealed that honeysuckle berries possess greater health-promoting properties than other berries which are widely consumed [34]. The study also showed that supplementation of apple juice with a honeysuckle berry had an impact on vitamin C, polyphenol and anthocyanin content, and significantly increased antioxidant activity. A combination of apple juice and a honeysuckle berry extract has also been used in the presented research. 

Although a number of research studies have focused on polyphenol-rich smoothies, none of them analysed the product enriched by purified extracts, free of organic acids and sugars, but showing a high concentration of not only phenolic compounds but also iridoids, making it a unique compound.

Therefore, the purpose of the study was to analyse the bioactivity of smoothies based on commonly consumed fruit enriched with a honeysuckle berry extract. The influence of the dose of the latter on the chemical composition, content of bioactive substances, antioxidant activity and sensory qualities of prepared smoothies has been assessed.

## 2. Materials and Methods

### 2.1. Instruments and Reagents

2,2-Diphenyl-1-picrylhydrazyl radical (DPPH); 6-hydroxy-2,5,7,8-tetramethylchroman-2-carboxylic acid (Trolox); 2,4,6-tri(2-pyridyl)-s-triazine (TPTZ), dimethyl sulfoxide (DMSO), FeCl3, and formic acid were purchased from Sigma-Aldrich (Steinheim, Germany). Acetic acid and Folin–Ciocalteu reagent were received from Chempur (Piekary Śląskie, Poland). Acetonitrile for high pressure liquid chromatography (HPLC- PDA) was obtained from POCh (Gliwice, Poland). All reagents were of analytical grade. Panzym Be XXL (Begerow GmbH & Co., Darmstadt, Germany) was used for depectinisation. An Amberlite XAD-16 resin column (Rohm and Hass, Chauny Cadex, France) and Alpha 1–4 LSC, Christ, Germany lyophilizer were used for dry extract preparation. A Thermomix TM 3 device (Vorwerk & Co. KG, Wuppertal, Germany) and hydraulic press (SRSE, Warsaw, Poland) were used for juice preparation. A digital refractometer (Atago RX-5000, Atago Co. Ltd., Saitama, Japan) was used for soluble solids analysis. A Brookfield DV-II + Pro viscometer (spindle number 63) was used for viscosity measurements. A centrifuge MPW-350 (MPW Med Instruments, Warsaw, Poland) was used for fluid separation. A UV-2401 PC spectrophotometer (Shimadzu, Kyoto, Japan) was used for UV/VIS analysis. A Dionex (Germering, Germany) device equipped with the diode array detector model Ultimate 3000, quaternary pump LPG-3400A, autosampler EWPS-3000SI, thermostatted column compartment TCC-3000SD, and controlled by Chromeleon v.6.8 software (Thermo Scientific Dionex, Sunnyvale, CA, USA) was used for HPLC-PDA analysis. The column used for the analysis was Cadenza Imtakt column CD-C18 (75 × 4.6 mm, 5 μm).

### 2.2. Plant Material

Honeysuckle berries (*Lonicera caerulea* L. var. *kamtschatica* Sevast.) of mixed cultivars were used for this study. The fruit was collected in 2017 during the growing season (namely May), frozen and stored at −80 °C until the juice was pressed and extracts were prepared. The fruit was harvested in Skierniewice (51°57′ N, 20°10′ E) in Poland. Black chokeberry fruit (*Aronia melanocarpa*) was collected during September 2017 in Stargard, Poland (53°20′ N, 15°02′ E). The strawberry (*Fragaria ananassa*) cultivar ‘Roxana’ was harvested in August 2017 in Kołomąć, Poland (53°52′ N, 15°07′ E) and apples of the cultivar ‘Champion’ were bought in Wrocław, Poland (51°06′ N, 17°01′ E) and further processed into juice with the addition of 5% rhubarb bought in the same place.

### 2.3. Ingredients for the Product Formulation

The designed product form was a smoothie. In order to obtain an attractive flavour and enhance the bioactivity, the following components were chosen for the smoothie mixture: strawberry fruit, chokeberry fruit, apple juice, and a honeysuckle berry extract (Table 1).

### 2.4. A Honeysuckle Berry Extract Preparation

A detailed extraction method was previously described by Szołtysik et al. [35]. Frozen fruit of honeysuckle berry (5 kg) was heated for 5 min at 95 °C in the Thermomix and shredded. The pulp was cooled down to 40 °C and depectinised in a water bath at 50 °C for 2 h by the addition of Panzym Be XXL (0.5 mL/1 kg). Further, juice was pressed from the prepared pulp using a hydraulic press. It was then filtered and run through a resin column to remove organic acids and sugars. Polyphenols and iridoids were eluted using 80% ethanol. In order to concentrate the eluate, a vacuum at 40 °C was used. The solvent was evaporated and lyophilized, resulting in a dry extract which was further added to the smoothies, according to the formulation quoted in Table 1.

### 2.5. Experiment Design

**Figure 1:**
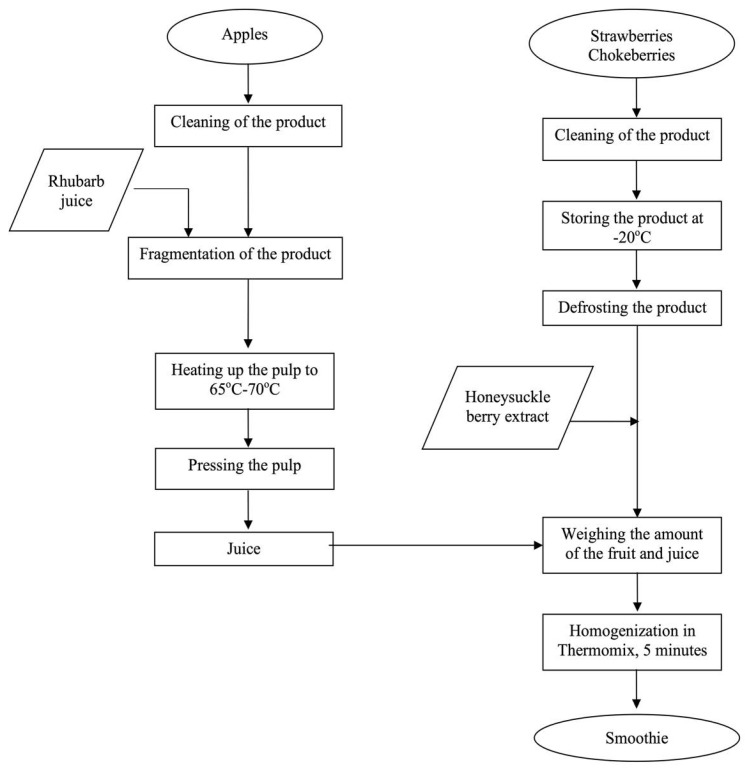
Scheme of the smoothie production.

The smoothies were prepared according to the method given in Figure 1.

### 2.6. Extraction of Compounds for Analysis

Frozen fruits of chokeberry and strawberry were homogenized, and 5 g of the product was dissolved in 40 g of 80% aqueous methanol acidified with 1% HCl and ultrasonicated for 20 min. The samples were stored for 24 h at 4 °C and ultrasonication was repeated. The extract was centrifuged at 19,000× g for 10 min. Both fruit extracts were diluted with re-distilled water; for chokeberry extract, at a ratio of 1:19 and for strawberry, 1:3, *v*/*v*. For HPLC-PDA analysis, the original extracts were also diluted with re-distilled water at a ratio of 1:1, *v*/*v*, and filtered through a hydrophilic PTFE 0.45 μm membrane (Millex Samplicity Filter, Merck, Germany).

### 2.7. Biochemical Composition and Physical Parameters

The biochemical compositions of prepared smoothies were analysed in accordance with European Standards (PN-EN). The soluble solid content, dry matter, and titratable acidity were analysed according to PN-EN 12,143:2000, PN-EN 12,145:2001, and PN-EN 12,145:2000, respectively. The soluble solid content was measured using a digital refractometer at room temperature. Dry matter was determined by combining the sample with diatomaceous earth, pre-drying and re-drying at reduced pressure. Pectin content was analysed in accordance with the Morris method described by Pijanowski et al. [36]. Ash, vitamin C, sugars and organic acid content analyses were performed according to the method previously described by Wojdyło et al. [37]. The organic acid content was measured using high-pressure liquid chromatography with an evaporative light-scattering detector (HPLC-ELSD). The measurements were carried out in triplicate. Viscosity was measured according to a procedure reported by Kucharska et al. [38]: rotation 10, time 30 s.

The content of total phenolics was analysed by the Folin–Ciocalteu method using gallic acid (GA) as a standard for the calibration curve; 0.1 mL of the sample was mixed with 0.2 mL of Folin-Ciocalteu reagent and 2 mL of water in 4 mL cuvettes. After 3 min, 1 mL of 20% sodium carbonate was added. The samples were incubated in the dark at room temperature for 1 h, and the absorbance was read at 765 nm using a spectrophotometer. The analysis was performed in triplicate and results were expressed as milligrams of GA equivalent (GAE) per 100 g fresh weight (FW).

### 2.8. Quantification of Polyphenols and Iridoids by HPLC-PDA

The method was previously described by Kucharska et al. [28]. The mobile phase consisted of a solvent A (4.5% aq. formic acid, *v*/*v*) and solvent B (100% acetonitrile). The elution system was as follows: 0–1 min 5% B in A, 20 min 25% B in A, 21 min 100% B, 26 min 100% B, and 27 min 5% B in A. The mobile phase flow rate was 1.0 mL/min, and the injection volume was 20 μL. The operation temperature was set to 30 °C. The particular compounds were detected at given wavelengths: iridoids were detected at 245 nm, flavan-3-ols at 280 nm, flavonols at 280 and 360 nm, phenolic acids and their derivatives at 320 nm, anthocyanins at 520 nm.

Iridoids were expressed as mg of loganic acid equivalents (LAE) per 100 g (FW), anthocyanins as cyanidin 3-*O*-glucoside equivalents (CygE) per 100 g FW, flavonols as quercetin 3-*O*-glucoside equivalents (QgE) per 100 g FW, phenolic acids as mg of 5-*O*-caffeoylquinic (chlorogenic) acid equivalents (CQAE) per 100 g FW, flavan-3-ols as catechin equivalents (CaE) per 100 g FW. Solutions of standards (1 mg/mL) were dissolved in 1 mL of methanol. The given amounts of stock solutions were diluted with 50% aqueous methanol (*v*/*v*) acidified with 1% HCl in order to obtain standard solutions.

### 2.9. Antioxidant Activity

The total antioxidant activity of the samples was determined using a ferric reducing antioxidant power ability of plasma (FRAP) assay according to Zhang et al. [39]. The solution was prepared by mixing acetate buffer (300 mM, pH 3.6), a solution of 10 μM TPTZ in 40 mM HCl, and 20 mM FeCl_3_ at 10:1:1 (*v*/*v*/*v*). The method for analysis of radical scavenging activity of the samples was previously described by Yen and Chen [40]. DPPH (100 μM) was dissolved in 96% ethanol. All measurements were performed in triplicate using a spectrophotometer. The absorbance was determined after 10 min at 593 nm for FRAP and at 517 nm for DPPH. A standard curve was prepared using different Trolox concentrations. Calibration curves, in the range of 0.01–5.00 μmol Trolox L^−1^, were applied for the quantification of the mentioned measurement of antioxidant activity. The dilution was taken into account while calculating the results, and they were expressed in μmol Trolox equivalent (TE) per 100 g FW. Additionally, antioxidant activity was determined using the ABTS assay described previously by Re et al. [41].

### 2.10. Sensory Analysis

The smoothies were subjected to a sensory analysis according to the method described by Wyspiańska et al. [42] using a 5-point hedonic scale, where 5 corresponded to excellent, 4 to very good, 3 to good, 2 to bad, and 1 to very bad. Sixteen assessors fulfilling the basic requirements of PN-ISO 3972 [43] were selected for the panel. The chosen quality descriptors were colour, consistency, odour, and flavour. The analysis was executed according to the principles established by the Declaration of Helsinki. Protocols protecting the rights and privacy of all participants were executed. Verbal consent of the participants was obtained.

### 2.11. Statistical Analysis

The results were analysed using the Statistica version 12.0 (StatSoft, Tulsa, OK, USA). One-way analysis of variance (ANOVA) by Duncan’s test was applied in order to compare the mean values. Differences were considered significant at α = 0.05.

## 3. Results and Discussion

### 3.1. Quantitative Analysis, and Total Flavonoids and Iridoids of Smoothie Components

The results of quantitative identification of total phenolics and total flavonoid compounds present in fruit used for smoothie production—chokeberries, strawberries, apple juice with 5% rhubarb juice, a honeysuckle berry extract—are shown in Table 2 together with the data reported in various literature sources. The compounds were analysed by their HPLC retention times, UV-vis spectra and by comparison to the literature data. Phenolic compounds (anthocyanins, flavan-3-ols, phenolic acids, flavonols) and monoterpenes (iridoids) were quantified.

The data collected show that chokeberries and a honeysuckle berry extract are the richest sources of analysed bioactive compounds. Based on the data obtained, the content of phenolics in a dry extract from honeysuckle berry is on average 19 times higher than that observed in chokeberries, 231 times higher than in strawberries, and 308 times higher than in apple juice. Some of the compounds might differ from the given literature values due to losses occurring in technological processes. Polyphenols’ stability can be affected by numerous factors, including pH, light, oxygen, enzymes, and thermal processes [53]. Taking into account flavan-3-ols, procyanidins and catechins are the most unstable among all phenolics and are readily oxidized, condensed, polymerized and reactive with other plant constituents [54]. Anthocyanins are responsible for the red, purple and blue colour; therefore, their amount might differ depending on factors such as cultivar or ripening stage of the fruit [55]. Iridoids are only found in a couple of fruits, such as honeysuckle berries, cornelian cherry fruit, cranberries, and bilberries [56].

### 3.2. Identification and Quantitative Analysis of the Phenolic Compounds and Iridoids of Smoothie Components and Smoothies

Table 3, Table 4, Table 5, Table 6 and Table 7 show the results of quantitative analysis of anthocyanins, flavonols, phenolic acids, flavan-3-ols and iridoids in all smoothie components and smoothies.

The abundance of phenolics and iridoids in the smoothies depended on the quantity and the origin of the ingredient used. The final products contained around 40% of an apple juice, 42% of strawberries, 18% of chokeberries and between 0.25–0.50% of a honeysuckle berry extract, which corresponded to the amount of bioactive compounds in the smoothies. The content of apple juice, being the liquid base of smoothies, was chosen to vary where a honeysuckle berry extract was added.

Both chokeberries and strawberries contained four different anthocyanins. Chokeberries were especially rich in cyanidin 3-galactoside (349.03 mg/100 g FW) and cyanidin 3-arabinoside (161.72 mg/100 g FW). Strawberries did not contain high concentrations of anthocyanins (24.27 mg/100g FW), compared to chokeberry (539.19 mg/100 g FW) or a honeysuckle berry extract (8808.30 mg/100 g FW). The main anthocyanin found in strawberries was pelargonidin 3-glucoside (21.04 mg/100 g FW). Apple juice did not contain any anthocyanins. As previously documented, a honeysuckle berry is a great source of various phenolics, including anthocyanins [56]. Anthocyanins are the most abundant compound in honeysuckle berry fruit, ranging between 36 and 51% of all phenolics [57]. The six out of 11 anthocyanins identified in all fruit together were found in the honeysuckle berry extract, while in chokeberries and strawberries only four were found. Cyanidin 3-glucoside was the major anthocyanin found in the honeysuckle berry extract (7433.40 mg/100 g FW). The mentioned anthocyanins, together with cyanidin 3-galactoside, were dominant ones in smoothies 2 and 3. Smoothie 1 contained only 2.29 mg/100 g FW of cyanidin 3-glucoside, and the amount significantly increased in the case of Smoothie 2 and Smoothie 3 (23.75 and 102.78 mg/100 g FW, respectively). A significant difference between smoothies 2 and 3 was additionally observed in the case of cyanidin 3,5-diglucoside, pelargonidin 3-glucoside and peonidin 3-glucoside. Cyanidin-3-*O*-sophoroside, cyanidin 3-rutoside, pelargonidin 3-glucoside and peonidin 3-rutoside were found in the extract but were not found in the smoothies. They possibly might react with other compounds or were degraded during smoothie preparation. The addition of a honeysuckle berry extract resulted in a significant difference in total anthocyanin content between all of the smoothies. In fruit smoothies developed by Nowicka et al. [58], the anthocyanins were found to be within the range of 23.59 mg/100 g FW to 189.02 mg/100 g FW. Anthocyanins cause the red fruit colour and possess numerous health-promoting properties [59]. Anthocyanins were proved to show cardioprotective, antitumor, vasodilatory and glucoregulatory effects [60,61,62]. Prevention of hyperglycaemia and thus the anti-diabetic properties of anthocyanins are based on the ability to reduce lipid absorption [63]. Cyanidin 3-glucoside, which was highly concentrated in smoothies 2 and 3, plays a crucial role in counteracting diabetes [58]. The compound shows a positive impact on insulin resistance by influencing transcription factor forkhead box O1 [63].

Single flavonols were not found in high concentrations in the smoothies (not exceeding 15.34 mg/100 g FW) compared to other phenolics. In this study, five flavonols were found in the smoothies; however, a total of eight types of flavonols were found in all used fruit combined. Quercetin 3-*O*-galactoside, quercetin 3-*O*-arabinoside and quercetin 3-*O*-xyloside were undetectable in the smoothies, because of their occurrence at very low concentrations (below 1 mg/100 g) and were only found in the apple juice. Quercetin 3-*O*-glucoside was the only flavonol found in all the tested fruit. In strawberries, only quercetin 3-*O*-glucoside and quercetin 3-*O*-glucuronide were found, in concentrations of 2.67 and 1.49 mg/100 g FW, respectively. In apple juice, flavonols ranged between 0.28 and 1.10 mg/100 g FW, which included quercetin 3-*O*-rutoside, quercetin 3-*O*-glucoside, quercetin 3-*O*-galactoside, quercetin 3-*O*-arabinoside and quercetin 3-*O*-xyloside. Chokeberries and the honeysuckle berry extract were the richest sources of flavonols. In chokeberries, quercetin-*O*-dihexoside, quercetin 3-*O*-vicianoside, quercetin 3-*O*-rutoside and quercetin 3-*O*-glucoside were identified, and their concentrations ranged from 2.06–13.49 mg/100 g FW. The flavonols in a honeysuckle berry extract were much higher: between 315.01 and 1792.40 mg/100 g FW. Quercetin 3-*O*-vicianoside, quercetin 3-*O*-rutoside and quercetin 3-*O*-glucoside were identified in the extract. The dominant flavonol in all of the smoothies was quercetin 3-rutoside. Quercetin is ubiquitously distributed throughout the plant kingdom [64]. It shows an antiviral potential and was recently reported to be the most effective natural compound against influenza [65]. A recent study also reported a therapeutical effect of quercetin against COVID-19 [66]. The total content of flavonols was significantly different in all of the smoothies and ranged between 7.27 (Smoothie 1) and 26.26 mg/100 g FW (Smoothie 3). In another study, where smoothies with sea buckthorn were analysed [67], the concentration of flavonols in smoothies ranged between 25.46 mg/100 g FW and 65.26 mg/100 g FW, depending on the ingredients added. Flavonols exert functional effects in numerous cellular targets playing a role in carcinogenesis [65]. Additionally, they are known for their antibacterial action, possibly via cell walls and membrane damage and ATP metabolism [65].

A total of six phenolic acids were found in the smoothie ingredients, while three of them were present in the final product. Low concentrations of phenolic acids were found in strawberries and apple juice, at 5.69 and 7.02 mg/100 g FW, respectively. In strawberries, only p-coumaryl-beta-d-glucose was present; however, its concentration was as low as 5.62 mg/100 g FW, and it was not found in the final product. Similarly, p-coumaroylquinic acid found in apple juice at a concentration of 2.39 mg/100 g FW was not identified in the smoothies. Chlorogenic acid was another phenolic acid in apple juice (4.70 mg/100 g FW). In chokeberries, neochlorogenic and chlorogenic acids were identified, and their total concentration was 72.41 mg/100 g FW, being significantly higher than the acid concentration in all the smoothies, apple juice and strawberry. A honeysuckle berry extract was rich in neochlorogenic (304.96 mg/100 g FW), chlorogenic (2324.25 mg/100 g FW), and two dicaffeoylquinic acids (114.14 mg/100 g FW and 412.63 mg/100 g FW), and the total acid content was equal to 3155.98 mg/100 g FW. In Smoothie 1, only neochlorogenic (6.65 mg/100 g FW) and chlorogenic acids (11.93 mg/100 g FW) were identified. In smoothies with the honeysuckle berry extract, both of these acids were also present; however, their concentrations were significantly higher than that of Smoothie 1. Additionally, Smoothie 2 and Smoothie 3 contained a dicaffeoylquinic acid isomer originating from the honeysuckle berry extract. The total content of phenolic acids in all the smoothies was significantly different. Phenolic acids are the second most abundant phenolics found in honeysuckle berry fruit and contribute around 24% of total polyphenols [57]. Phenolic acids are associated with antimutagenic, chemopreventive, cardioprotective and probiotic effects [68]. Chlorogenic acid, which was the most abundant acid in all the smoothies, lowers liver glucose synthesis by activating adenosine monophosphate-activated protein kinase, having a positive effect on Type 2 diabetes mellitus [68]. The mentioned acid is able to suppress the expression of cyclooxygenase-2 and inducible nitric oxide synthase related to osteoarthritis. Moreover, phenolic acids inhibit angiogenesis and also inhibit enzymes related to Alzheimer’s disease. They are able to decrease acetylcholine and butyrylcholine breakdown in the brain.

The richest source of flavanols was the honeysuckle berry extract (1200 mg/100 g FW), while they were not found in chokeberry. In strawberries, procyanidin B1, catechin, procyanidin B2 and epicatechin were detected. All the mentioned flavanols were found in apple juice; however, their concentrations were significantly lower than those observed for strawberry. Additionally, procyanidin C1 (3.17 mg/100g FW) was present in the apple juice. The honeysuckle berry extract was especially rich in procyanidin C1 (1034.66 mg/100g FW), followed by procyanidin B1 (130.24 mg/100g FW) and catechin (35.10 mg/100g FW). The total content of flavanols was significantly different in all the smoothies and ranged between 13.42 and 28.76 mg/100g FW. Smoothies did not contain epicatechin and procyanidin C1, and the most abundant was procyanidin B1 (6.39–14.43 mg/100g FW). The lack of some of the flavanols in the smoothies could be caused by the low amounts of these compounds in the fruit (between 3.17 and 16.53 mg/100g FW) and thus they were too low in the final product to be observed. Flavanol-rich food shows positive health impacts, e.g., improvement of insulin sensitivity, reduced platelet aggregation, recovery of endothelial function, decreased blood pressure. The average flavanol intake by an adult has been estimated to be between 50 and 100mg/day, corresponding to approximately a glass of Smoothie 3.

Iridoids are rarely found in fruit, and a honeysuckle berry is one of the exceptional sources of them [56]. In the smoothie with no supplementation of the honeysuckle berry extract, no iridoids were detected. In the honeysuckle berry extract, the main iridoid was sweroside, at 3825.96 mg/100g FW, while an isomer of loganic acid was detected in the lowest amount, at 317.46 mg/100g FW. The amount of the latter was too low to be found in the final products. The reason for such a low concentration of loganic acid is the removal of acids and sugars during the dry extract preparation and separation in the resin column. The content of all iridoids in Smoothie 3 was significantly higher than in Smoothie 2; therefore, the addition of honeysuckle berries can serve as a rich source of this unique compound. The two dominant iridoids in smoothies with the honeysuckle berry extract were sweroside and loganin. Sweroside (found in the highest concentration) protects from myocardial ischemia by inhibiting apoptotic cascades and prevents non-alcoholic steatohepatitis by decreasing the synthesis of caspase-1 and interleukin-1 β [69]. Iridoids result in the bitter taste of the fruit and have been found to contribute to numerous antioxidant and anti-inflammatory properties [70,71]. Moreover, they show a neuroprotective effect, including prevention of Alzheimer’s disease, as well as a hepatoprotective effect, including antifibrotic properties [30]. Iridoids also act as anticonvulsants, show a sedative effect and could minimize irritable bowel syndrome.

The most abundant in all the smoothies were anthocyanins (109.28–253.86 mg/100g FW) followed by iridoids, highly concentrated in Smoothie 2 and Smoothie 3 (49.36 and 177.79 mg/100g FW, respectively). The highest values of flavonols, anthocyanins, phenolic acids, flavan-3-ols and iridoids were all recorded for Smoothie 3. Therefore, Smoothie 3 shows the highest health potential, including neuro- and cardioprotective, anti-inflammatory, anti-cancer and anti-diabetic effects associated with the presence of polyphenols and iridoids [72].

The honeysuckle berry extract shows a significantly higher content of bioactive compounds than other smoothie components. Similarly, the smoothie with the highest addition of the extract contained higher concentrations of the analysed compounds than the other two. Some compounds are not present in the final product, which might be the effect of pre-treatment or the processing method of the components used. The lack of a direct dependency between the sum of smoothie components and the total content of polyphenols in smoothies might be the result of partial degradation of the compounds during the technological process or possible conversion of them. Polyphenols are very unstable, highly reactive, and are prone to transformations into various products during post-harvesting and processing [53]. As Michalska and Łysiak [73] have reported, mechanical and chemical processing negatively affects phenolic content. Polyphenols are highly prone to degradation and reaction with other food constituents [74]. The stability of phenolics can be affected by structural changes resulting from the impact of light, pH, temperature, interactions or metal ions [53]. When it comes to temperature, anthocyanins are susceptible to degradation at high temperatures, contrary to phenolic acids being much more stable in such conditions. Moreover, compounds like ascorbic acid and sugars are claimed to affect polyphenol stability [75]. Phenolic compounds could be hydrolysed to aglycones [76]. They are also vulnerable to heat. Anthocyanins may undergo changes involving polymerization or degradation to hydroxybenzoic acids. Flavan-3-ols are prone to conversion to tannins. Therefore, properties of obtained products might differ from those of their precursors. On the other hand, polyphenolic compounds show the ability of synergistic activity, unlike other bioactive compounds [77].

The results present a rich content of diverse bioactive compounds in the smoothies, which further corresponds to their bioactive properties.

### 3.3. Antioxidant Activity

The antioxidant activity DPPH, ABTS and FRAP of the fruit used for smoothie production is presented in Table 8. In the entire analysis, the lowest activity was reported for apple juice, followed by strawberry and chokeberry, while the highest values were reported for the honeysuckle berry extract. The results recorded in the case of the honeysuckle berry dry extract are from seven to 61 times higher than for the other fresh fruit, depending on the method used. Smoothie 3 showed significantly higher antioxidant activity than Smoothie 1 and Smoothie 2 despite the testing method used. The activity measured for the smoothie with no extract was significantly lower than the one with 0.25% extract addition, in the case of DPPH and FRAP. The smoothie with the highest content of the honeysuckle berry extract showed more than double results for each of the performed tests than the smoothie with no extract. As the two variables were the concentration of the honeysuckle berry dry extract and the apple juice content, which had the lowest antioxidant activity, the high results of Smoothie 3 can be associated with the bioactivity of the honeysuckle berry extract.

The antioxidant activity of other smoothies reported in the literature by Nowicka et al. [58] showed ABTS results for diverse smoothies with *Prunus* fruit ranging from 23.5 to 52.1 μmol TE/g. In the case of the smoothies analysed in this research, the ABTS values ranged from 31.32 to 63.65 μmol TE/g. Other research showed FRAP results for a home- made smoothie made of pear, apple and kiwifruit equal to 3.12 μmol TE/g and for a commercial smoothie from the same products equal to 13.74 μmol TE/g, while the values presented in this study range between 18.80 and 41.92 μmol TE/g [78]. Compared to a strawberry cloudy juice, for which ABTS values ranged between 0.69 and 0.89 μmol TE/mL depending on the variety used, the results obtained for smoothies with the honeysuckle berry extract are almost a hundred times higher [79]. A strawberry puree ABTS result showed values between 2.00 and 2.49 μmol TE/mL, which is much lower than the smoothie values in this research. Another study reported DPPH values for a pumpkin puree enriched with Japanese quince, cornelian cherries, strawberries and apples ranging from 1.02 to 3.79 μmol TE/g, while DPPH values for the smoothies presented below range from 12.28 up to 26.85 μmol TE/g [80]. Comparing the smoothies to strawberry jams enriched with either chokeberries, elderberries, Japanese quince, flax seeds or wheat germ, which showed an ABTS mean value equal to 74.00 μmol TE/g, the smoothie values presented in the study were lower [81].

The previous studies suggest that the content of phenolic compounds and their type are important factors influencing antioxidant properties [58,82,83]. The diverse polyphenol content of the fruit used for smoothie preparation corresponded to a high antioxidant activity having important health benefits. Therefore, the consumption of smoothies with the honeysuckle berry dry extract, particularly Smoothie 3, may lead to a reduction of free radicals and help counteract diseases like diabetes, hypertension, and cancer [84].

### 3.4. Sensory Evaluation of the Smoothies

The sensory quality is one of the most important factors having an impact on consumer decisions [85]. The results of consumer preferences depending on colour, consistency, aroma and flavour of the smoothies are presented in Table 9. The analysis showed that the more of the honeysuckle berry extract was added, the more the scores of quality descriptors decreased. Smoothie 3 with 0.5% w/w honeysuckle berry extract added resulted in a low acceptance by consumers, particularly in terms of taste, which could be associated with the tartness and bitterness of honeysuckle berries. The specific taste of the honeysuckle berry is associated with the quality and quantity of polyphenols, and the bitterness is determined by secoiridoids [28]. The statistical analysis indicated no significant difference between the individual quality descriptors of the smoothie with no extract added (Smoothie 1) and 0.25% w/w extract (Smoothie 2). Therefore, lower supplementation with the honeysuckle berry extract was still accepted by the consumers. Only the consistency evaluation showed no significant difference among all of the smoothies. The intense purple–red colour change in Smoothie 3, associated with the higher content of anthocyanins, was not attractive to the consumers. Similar results were obtained for aroma and flavour and were less attractive for the consumers than Smoothie 1 and 2. Honeysuckle berries are characterized as bitter to sour-sweet, and their high concentration in smoothies did not meet consumer acceptance. As reported in other studies, consumer preferences are correlated with the sensation of balanced sweet and sour tastes, fruit flavour and sweetness [85].

Smoothie 3 including a 0.5% w/w extract was found to be the least attractive in terms of three out of four parameters; therefore, only Smoothie 1 and 2 were used for further analysis of physicochemical parameters and organic acid content.

### 3.5. Physicochemical Parameters and Organic Acids of the Smoothies

Smoothie 1 and Smoothie 2 were analysed in terms of chemical composition, including dry matter, ash content, viscosity, extract, acidity, pectin, sugar and vitamin C content (Table 10).

The dry matter content did not differ significantly between the two smoothies, reaching values between 13.10–13.16%. Similarly, the differences in ash content were not significant among the smoothies and ranged from 0.30–0.35%. In terms of viscosity, the higher value was observed for Smoothie 2 (2839 Pa·s). The parameter was affected by the addition of the honeysuckle berry extract. In terms of soluble solid content, no significant difference was found between Smoothie 1 and Smoothie 2. The titratable acidity values were similar for Smoothie 1 and 2 (0.73% and 0.74%, respectively).

Smoothies are mostly composed from widely consumed fruit, such as bananas and apples, but often red fruit such as blackberries and strawberries are added [9]. The previous studies reported the pectin content for blackberries in the range of 0.40–1.19%, for raspberries in the range of 0.10–0.88%, while the highest values were in apricots, 0.42–1.32% [86]. A study by Tkacz et al. [67] revealed that 100% sea buckthorn juice contained 0.61% pectin. Both smoothies analysed in the current study showed values of pectin ranging from 0.80 to 0.89%, making them a good source of dietary fibre. Pectins are proved to counteract obesity, diabetes and cardiovascular diseases [87].

Regarding vitamin C, its level was similar in both smoothies (14.02–14.12 mg/100 g). A slightly higher amount of vitamin C in raspberry sorbet was measured in an analogical study, at 20 mg/100 g [88]. The values observed in the same study were 60 mg/100 g for a strawberry sorbet and 10 mg/100 g for a bilberry sorbet. A recommended daily intake of vitamin C is 90 mg/day for men and 75 mg/day for women [89], which is equal to almost 2.5 glasses of Smoothie 1 or Smoothie 2. A half cup of raw red pepper contains 95 mg of vitamin C [90], showing that the presented smoothies are not a very rich source of this vitamin.

The analysed sugar content ranged between 8.34 and 8.73%. Similar outcomes were presented in another study analysing two smoothies produced from cherry juice combined with a peach and an apricot puree, and its content ranged between 8.19 and 9.86% [58]. The data presented regarding commercial smoothies showed the sugar content equal to 13% [91].

The analysis of total polyphenol content determined the substances reacting with the Folin–Ciocalteu reagent, thus providing indirect information about the antioxidant capacity. The results increased significantly with the addition of the honeysuckle berry extract and ranged from 346.95 to 426.81 mg GAE/100 g. The total polyphenol content reported for banana pulp varied from 38 to 128 mg GAE/100 g, depending on the cultivar [92]. Smoothies composed from strawberries, whole apples, bananas, orange and apple juice showed polyphenol content equal to 44 mg GAE/100 mL. The results measured for the smoothie with the honeysuckle berry extract are almost 10 times higher. In a study by Ribeiro et al. [93], the measured bioaccessibility of phenolic compounds from smoothies differed from 20 to 47% between gastric and intestinal digests, showing high bioavailability of these phenolics.

Apart from the viscosity and polyphenol content, the other parameters did not show significant discrepancies between the smoothies. Compared to other studies, the analysed smoothies could help counteract metabolic diseases due to a high pectin content and enhance the immune system, associated with the presence of vitamin C [90]. The higher polyphenol content of the smoothie with honeysuckle berry extract confirms earlier results showing high levels of phenolic compounds in the extract, corresponding to important biological activities such as anticancer, anti-inflammatory and anti-microbial effects [94].

The organic acid content in the smoothies is presented in Table 11. Among all the acids, quinic, citric, malic, shikimic and oxalic acids were identified. The most abundant one was quinic acid, followed by malic, citric and shikimic acids. The oxalic acid was only present in a trace amount. The level of analysed acids was not significantly different between the smoothies, which is associated with the addition of the honeysuckle berry extract being purified from organic acids and sugars. The dominant one in all smoothies was quinic acid, and the recorded values ranged from 0.840 g/100 g FW for Smoothie 1 to 0.868 g/100 g FW for Smoothie 2. Quinic acid shows numerous health-promoting properties, such as hepatoprotective, choleretic, antibacterial, antiviral, and anti-inflammatory activities [95]. The lowest concentration identified was of oxalic acid, with values in the range of 0.005–0.006 g/100 g FW. Another study focused on smoothie analysis reported values of quinic acid ranging between 0.65–1.38 g/100g FW, and oxalic acids between 0.05–0.18 g/100g FW [96]. A high concentration of oxalic acid is associated with decreased mineral bioavailability and formation of calcium oxalates in urinary stones; therefore, decreasing its amount is highly desired. The conducted studies prove that sweet taste and flavour depend not only on a high sugar content but also on a low concentration of acids [97]. The level of sugars and organic acids in the products determine consumer perception and thus acceptance. As was also presented in Table 10, the sugar content and titrable acidity of both smoothies were similar and thus equally accepted by the consumers.

## 4. Conclusions

The presented study provides comprehensive insights into the properties, composition, and sensory perception of different smoothies with the honeysuckle berry polyphenol-iridoid dry extract. The scope of the analyses included the content of bioactive compounds (polyphenols, iridoids, organic acid, biochemical composition), antioxidant activity and sensory analysis performed by the consumers.

The analysed smoothies varied significantly in many characteristics, and the main factor affecting the variation appeared to be the content of honeysuckle berry extract in the final product. The honeysuckle berry extract shows significantly higher concentrations of all the phenolics and iridoids than other smoothie components. Addition of the extract resulted in a higher content of bioactive compounds and significantly increased the antioxidant activity of the smoothies. The highest level of flavonols, anthocyanins, phenolic acids, flavan-3-ols, iridoids were all recorded for the smoothie with the highest honeysuckle berry extract content. Smoothie 2 and Smoothie 3 were particularly rich in anthocyanins and iridoids. Highly concentrated in these two smoothies, cyanidin 3-glucoside plays an important role in counteracting diabetes. The main iridoid found in the smoothies with the extract was sweroside, which is known to protect against myocardial ischemia and non-alcoholic steatohepatitis.

Compared to other products having a similar consistency, like juices, purees or other smoothies, the smoothies with the honeysuckle berry extract show a few-fold higher antioxidant capacity. The high antioxidant activity of the extract and of smoothies to which it is added is the result of a rich content of bioactive compounds. Smoothie 3 showed the strongest antioxidant activity and the highest total content of polyphenols as well as iridoids which rarely occur in fruit but show a wide range of health properties. Hence, this smoothie shows the greatest health potential, such as cardioprotective, anti-inflammatory, anti-viral, anti-cancer and anti-diabetic effects. However, iridoids are also associated with the bitter taste, which is not widely accepted by the consumers. The sensory analysis showed that the product with the highest honeysuckle berry concentration (0.5%/w) scored less than the ones with lower concentration (0.25%/w) and no extract. Therefore, a smoothie with a high potential for the market is Smoothie 2, which is attractive in terms of a taste, promotes honeysuckle berry consumption and shows a high content of bioactive compounds and antioxidant activity, and thus a high health potential. The smoothie with the honeysuckle berry extract did not differ significantly from Smoothie 1 in terms of biochemical composition, e.g., titrable acidity, sugar, organic acids and pectin content, but showed a higher total polyphenol content, which is an important aspect in health promotion.

For an individual consumer, the study serves as a way to meet the recommendation to consume 400 g of fruit per day [98] and a recipe for delivering antioxidants and protecting against diseases by introducing honeysuckle berry to the everyday diet in a convenient form of smoothie. As only a few products with a honeysuckle berry have been introduced into the market, there is a great potential for smoothies with the extract to become widely available for consumers. The unique features of smoothies with the honeysuckle berry extract provide an interesting topic for further research. This study showed the initial balance between the concentration of the honeysuckle berry extract and a high bioactivity while maintaining overall consumer acceptance. The data could serve as an important basis for designing an innovative product with health-promoting properties using honeysuckle berry, which still remains an undervalued fruit. Further in vivo studies confirming the health potential of the presented smoothies, focused on anti-inflammatory, anti-diabetic, anti-cancer effects, could be conducted.

## Figures and Tables

**Table 1:** Ingredients for the formulation of smoothie products.

Smoothie	Components	Mass [g]	Percentage Content [%]
Smoothie 1	Apple juice with 5% rhubarb juice	200.00	40.00
	Strawberry fruit	210.00	42.00
	Chokeberry fruit	90.00	18.00
Smoothie 2	Apple juice with 5% rhubarb juice	198.75	39.75
	Strawberry fruit	210.00	42.00
	Chokeberry fruit	90.00	18.00
	Honeysuckle berry extract	1.25	0.25
Smoothie 3	Apple juice with 5% rhubarb juice	197.50	39.50
	Strawberry fruit	210.00	42.00
	Chokeberry fruit	90.00	18.00
	Honeysuckle berry extract	2.50	0.50

**Table 2:** Content of phenolic compounds and iridoids in fresh components (chokeberry, strawberry, apple juice) (mg/100 g FW) and in a honeysuckle berry extract (mg/100 g dw (dry weight)) used for smoothie production.

Compound Group	Chokeberry Fruit	Literature Values	Strawberry Fruit	Literature Values	Apple Juice	Literature Values	Honeysuckle Berry Extract	Literature Values
Anthocyanins	539.19	300.00–600.00 [44,45]	24.27	8.50–65.90 [46]	N.D.^1^	0.00–124.60 [47,48]	8808.30	L.D.^2^
Flavonols	34.93	18.31 [49]	4.16	1.10–3.80 [46]	2.98	1.67 [47]	2397.20	L.D.
Phenolic acids	72.411	287.30–487.40 [50]	5.62	1.29 [51]	7.09	119.74 [47]	3155.98	L.D.
Flavan-3-ols	N.D.	0.78 [52]	56.55	3.39 [51]	29.34	140.67 [47]	1200.00	L.D.
Iridoids	N.D.	L.D.	N.D.	N.D.	N.D.	N.D.	21460.79	L.D.

^1^ N.D.—not detected; ^2^ L.D.—lack of data.

**Table 3:** Anthocyanin content (mg/100 g FW) of smoothie components and smoothies.

Source	cy 3,5-diglu ^1^	cy 3-soph	cy 3-gal	cy 3-glu	cy 3-rut	cy 3-ara	pg 3-glu	pg 3-rut	pn 3-glu	cy 3-xyl	pn 3-rut	pn 3-mal-glu	Total Content
Chokeberry fruit	N.D. ^2^	N.D.	349.03 ± 38.67a ^3^	10.33 ± 2.50d	N.D.	161.72 ± 17.79a	N.D.	N.D.	N.D.	18.11 ± 1.91a	N.D.	N.D.	539.19e
Strawberry fruit	N.D.	N.D.	N.D.	0.51 ± 0.05d	N.D.	N.D.	21.04 ± 0.05a	0.74 ± 0.01	N.D.	N.D.	N.D.	1.98 ± 0.02a	24.27a
Apple juice	N.D.	N.D.	N.D.	N.D.	N.D.	N.D.	N.D.	N.D.	N.D.	N.D.	N.D.	N.D.	N.D.
Honeysuckle berry extract	232.05 ± 4.31a	369.02 ± 1.81	N.D.	7433.40 ± 2.69a	460.59 ± 2.56	N.D.	N.D.	N.D.	235.02 ± 4.98a	N.D.	78.22 ± 0.96	N.D.	8808.30f
Smoothie 1	N.D.	N.D.	64.02 ± 2.21b	2.29 ± 0.24d	N.D.	30.43 ± 0.86b	9.17 ± 0.16c	N.D.	N.D.	3.37 ± 0.12b	N.D.	N.D.	109.28b
Smoothie 2	N.D.	N.D.	67.24 ± 4.83b	23.75 ± 2.50c	N.D.	33.32 ± 2.46b	10.02 ± 1.13c	N.D.	N.D.	3.30 ± 0.39b	N.D.	0.91 ± 0.08b	138.54c
Smoothie 3	3.72 ± 1.52b	N.D.	82.57 ± 2.92b	102.78 ± 13.64b	N.D.	46.16 ± 5.82b	12.42 ± 1.30b	N.D.	1.64 ± 0.21b	4.57 ± 0.51b	N.D.	N.D.	253.86d

^1^ cy 3,5-diglu, cyanidin 3,5-*O*-diglucoside; cy 3-soph, cyanidin 3-O-sophoroside; cy 3-gal, cyanidin 3-galactoside; cy 3-glu, cyanidin 3-glucoside; cy 3-rut, cyanidin 3- rutoside; cy 3-ara, cyanidin 3-arabinoside; pg 3-glu, pelargonidin 3-glucoside; pg 3-rut, pelargonidin 3-rutoside; pn 3-glu, peonidin 3-glucoside; cy 3-xyl, cyanidin 3-xyloside; pn 3-rut, pelargonidin 3-rutoside; pn 3-mal-glu, pelargonidin 3-malonyl-glucoside; ^2^ N.D.—not detected; ^3^ Mean values ± standard deviation (SD) with different letters (a, b, etc.) within the same row are significantly different (*p* < 0.05).

**Table 4:** Flavonol content (mg/100 g FW) of smoothie components and smoothies.

Source	q-dihex ^1^	q 3-vic	q 3-rut	q 3-glu	q 3-gal	q 3-ara	q 3-gluc	q 3-xyl	Total Content
Chokeberry fruit	12.00 ± 0.97a ^2^	2.06 ± 0.16b	13.49 ± 1.35b	7.38 ± 0.78b	N.D.	N.D.	N.D.	N.D.	34.93f
Strawberry fruit	N.D. ^3^	N.D.	N.D.	2.67 ± 0.01c	N.D.	N.D.	1.49 ± 0.06a	N.D.	4.16b
Apple juice	N.D.	N.D.	1.10 ± 0.28b	0.35 ± 0.31c	0.28 ± 0.24	0.82 ± 0.10	N.D.	0.43 ± 0.06	2.98a
Honeysuckle berry extract	N.D.	315.01 ± 4.38d	1702.4 ± 18.56a	379.79 ± 4.12a	N.D.	N.D.	N.D.	N.D.	2397.20g
Smoothie 1	1.41 ± 0.16c	0.58 ± 0.05b	2.42 ± 0.09b	2.22 ± 0.03c	N.D.	N.D.	0.64 ± 0.66c	N.D.	7.27c
Smoothie 2	1.94 ± 0.11c	1.38 ± 0.11b	4.03 ± 0.91b	3.39 ± 0.08c	N.D.	N.D.	0.56 ± 0.01d	N.D.	11.30d
Smoothie 3	2.75 ± 0.11b	3.71 ± 0.52b	15.34 ± 1.92b	3.47 ± 0.47c	N.D.	N.D.	0.99 ± 0.13b	N.D.	26.26e

^1^ q-dihex, quercetin *O*-dihexoside; q 3-vic, quercetin 3-*O*-vicianoside; q 3-rut, quercetin 3-*O*-rutoside; q 3-glu, quercetin 3-*O*-glucoside; q 3-gal, quercetin 3-*O*-glucoside; q 3-gal, quercetin 3-*O*-galactoside; q 3-ara, quercetin 3-*O*-arabinoside; q 3-gluc, quercetin 3-*O*-glucuronide; q 3-xyl, quercetin 3-*O*-xyloside; ^2^ Mean values ± standard deviation (SD) with different letters (a, b, etc.) within the same row are significantly different (*p* < 0.05); ^3^ N.D.—not detected.

**Table 5:** Phenolic acid content (mg/100 g FW) of smoothie components and smoothies.

Source	3-CQA ^1^	5-CQA	p-coum-glu	di CQA 1	di CQA 2	p-coum	Total Content
Chokeberry fruit	33.10 ± 2.29b ^2^	39.31 ± 3.10b	N.D.	N.D.	N.D.	N.D.	72.41f
Strawberry fruit	N.D. ^3^	N.D.	5.62 ± 0.00	N.D.	N.D.	N.D.	5.62a
Apple juice	N.D.	4.70 ± 1.51f	N.D.	N.D.	N.D.	2.39 ± 0.38	7.09b
Honeysuckle berry extract	304.96 ± 1.69a	2324.25 ± 9.69a	N.D.	114.14 ± 2.70	412.63 ± 1.36a	N.D.	3155.98g
Smoothie 1	6.65 ± 0.69d	11.93 ± 1.02e	N.D.	N.D.	N.D.	N.D.	18.58c
Smoothie 2	7.69 ± 0.38cd	17.85 ± 0.94d	N.D.	N.D.	1.15 ± 0.20c	N.D.	26.69d
Smoothie 3	9.84 ± 2.07c	34.85 ± 4.21c	N.D.	N.D.	5.70 ± 0.71b	N.D.	50.39e

^1^ 3-CQA, neochlorogenic acid; 5-CQA, chlorogenic acid; p-coum-glu, p-coumaroyl-beta-d-glucose; di CQA 1, dicaffeoylquinic acid 1; di CQA 2, dicaffeoylquinic acid 2; p-coum, p-coumaroylquinic acid. ^2^ Mean values ± standard deviation (SD) with different letters (a, b, etc.) within the same row are significantly different (*p* < 0.05); ^3^ N.D.—not detected.

**Table 6:** Flavan-3-ols content (mg/100 g FW) of smoothie components and smoothies.

Source	PC B1 ^1^	(+) Cat	PC B2	(–) Epicat	PC C1	Total Content
Chokeberry fruit	N.D. ^2^	N.D.	N.D.	N.D.	N.D.	N.D.
Strawberry fruit	26.65 ± 0.19b ^3^	7.72 ± 0.03b	5.65 ± 0.10e	16.53 ± 0.17a	N.D.	56.55e
Apple juice	1.40 ± 0.03e	1.90 ± 1.31d	11.54 ± 2.21b	11.12 ± 1.13b	3.17 ± 0.39	29.13d
Honeysuckle berry extract	130.24 ± 6.90a	35.10 ± 1.27a	1034.66 ± 2.20a	N.D.	N.D.	1200.00f
Smoothie 1	6.39 ± 0.03d	2.43 ± 0.78d	4.60 ± 0.33f	N.D.	N.D.	13.42a
Smoothie 2	13.70 ± 1.69c	3.83 ± 0.60c	10.81 ± 0.11d	N.D.	N.D.	28.34b
Smoothie 3	13.43 ± 1.97c	4.23 ± 0.24c	11.10 ± 1.93e	N.D.	N.D.	28.76c

^1^ PC B1, procyanidin B1; (+)cat, (+)catechin; PC B2, procyanidin B2; (-)epicat, (-)epicatechin; PC C1, procyanidin C1; ^2^ N.D.—not detected; ^3^ Mean values ± standard deviation (SD) with different letters (a, b, etc.) within the same row are significantly different (*p* < 0.05).

**Table 7:** Iridoid content (mg/100 g FW) of smoothie components and smoothies.

Source	LA Isomer ^1^	LA	LAp	7-epi-LAp	S	Lo	pS	Total Content
Chokeberry fruit	N.D. ^2^	N.D.	N.D.	N.D.	N.D.	N.D.	N.D.	N.D.
Strawberry fruit	N.D.	N.D.	N.D.	N.D.	N.D.	N.D.	N.D.	N.D.
Apple juice	N.D.	N.D.	N.D.	N.D.	N.D.	N.D.	N.D.	N.D.
Honeysuckle berry extract	317.46 ± 0.91	3947.78 ± 3.76a ^3^	2462.14 ± 4.30a	2509.67 ± 5.00a	7828.65 ± 6.15a	3825.96 ± 5.74a	569.13 ± 0.24a	21460.79c
Smoothie 1	N.D.	N.D.	N.D.	N.D.	N.D.	N.D.	N.D.	N.D.
Smoothie 2	N.D.	6.56 ± 0.42c	4.59 ± 0.95c	4.25 ± 0.29c	14.09 ± 1.35c	19.87 ± 1.16c	N.D.	49.36a
Smoothie 3	N.D.	29.66 ± 2.65b	17.86 ± 0.75b	17.01 ± 2.45b	57.32 ± 5.75b	51.85 ± 10.09b	4.09 ± 0.80b	177.79b

^1^ LA isomer, loganic acid isomer; LA, loganic acid; LAp, loganic acid pentoside; 7-*epi*-LAp, 7-*epi*-loganic acid 7-*O*-pentoside; S, sweroside; Lo, loganin, pS- pentosyl sweroside; ^2^ N.D.—not detected. ^3^ Mean values ± standard deviation (SD) with different letters (a, b, etc.) within the same row are significantly different (*p* < 0.05).

**Table 8:** Antioxidant activity of fresh components, smoothies (μmol TE/g FW) and the extract (μmol TE/g dw) used for smoothie production.

Method	Chokeberry Fruit	Strawberry Fruit	Apple Juice	Honeysuckle Berry Extract	Smoothie 1	Smoothie 2	Smoothie 3
DPPH	45.49 ± 2.48b ^1^	10.22 ± 0.36f	2.12 ± 0.16g	1 230.23 ± 39.84a	12.28 ± 1.12e	14.30 ± 0.91d	26.85 ± 0.38c
ABTS	90.61 ± 8.72b	23.79 ± 1.06e	5.15 ± 0.40f	2 663.87 ± 49.11a	31.32 ± 1.72de	33.82 ± 1.24d	63.75 ± 3.63c
FRAP	60.97 ± 3.16b	14.66 ± 0.60f	3.12 ± 0.14g	2 450.80 ± 67.21a	18.80 ± 0.70e	23.72 ± 0.40d	41.91 ± 2.49c

^1^ Mean values ± standard deviation (SD) with different letters (a, b, etc.) within the same row are significantly different (*p* < 0.05).

**Table 9:** Results of five-point sensory analysis of smoothies.

Parameter	Smoothie 1	Smoothie 2	Smoothie 3
Colour	4.19 ± 0.66a ^1^	4.13 ± 0.81a	3.38 ± 1.45b
Consistency	3.94 ± 0.77a	3.88 ± 0.81a	3.50 ± 1.21a
Aroma	4.5 ± 0.52a	4.19 ± 0.91a	3.63 ± 0.96b
Flavour	4.13 ± 0.72a	3.88 ± 0.72a	2.19 ± 0.83b
Final score	4.19	4.02	3.17

^1^ Mean values ± SD with different letters (a, b, etc.) within the same row are significantly different (*p* < 0.05).

**Table 10:** Biochemical composition and physical parameter of the smoothies.

Parameters	Smoothie Type	
	Smoothie 1	Smoothie 2
Dry matter [%]	13.16 ± 0.19a ^1^	13.10 ± 0.28a
Ash [%]	0.35 ± 0.05a	0.30 ± 0.40a
Viscosity [Pa·s]	2543.00 ± 0.01a	2839.00 ± 0.01b
Soluble solid [°Bx]	11.90 ± 0.01a	12.00 ± 0.01a
Titratable acidity [%]	0.73 ± 0.01a	0.74 ± 0.02a
Pectin [%]	0.80 ± 0.60a	0.89 ± 0.08a
Vitamin C [mg/100g FW]	14.12 ± 0.35a	14.02 ± 0.20a
Sugar content [%]	8.34 ± 0.12a	8.73 ± 0.66a
Total polyphenol content [mg GAE/100g]	346.95 ± 20.70a	426.81 ± 20.69b

^1^ Mean values ± SD with different letters (a, b, etc.) within the same row are significantly different (*p* < 0.05).

**Table 11:** The concentrations of organic acids (g/100 g FW) of the smoothies.

Organic Acid	Smoothie 1	Smoothie 2
Quinic acid	0.840 ± 0.028a ^1^	0.868 ± 0.003a
Citric acid	0.337 ± 0.012a	0.348 ± 0.004a
Malic acid	0.332 ± 0.009a	0.355 ± 0.000a
Shikimic acid	0.120 ± 0.002a	0.130 ± 0.010a
Oxalic acid	0.005 ± 0.000a	0.006 ± 0.001a

^1^ Mean values ± SD with different letters (a, b, etc.) within the same row are significantly different (*p* < 0.05).

## Data Availability

The data used to support the findings of this study can be made available by the corresponding author upon request.
